# TLR7 Agonist GS-9620 Is a Potent Inhibitor of Acute HIV-1 Infection in Human Peripheral Blood Mononuclear Cells

**DOI:** 10.1128/AAC.01369-16

**Published:** 2016-12-27

**Authors:** Rujuta A. Bam, Derek Hansen, Alivelu Irrinki, Andrew Mulato, Gregg S. Jones, Joseph Hesselgesser, Christian R. Frey, Tomas Cihlar, Stephen R. Yant

**Affiliations:** Department of Biology, Gilead Sciences, Inc., Foster City, California, USA

**Keywords:** GS-9620, TLR7, antiviral agents, human immunodeficiency virus, interferons

## Abstract

GS-9620 is a potent and selective oral Toll-like receptor 7 (TLR7) agonist that directly activates plasmacytoid dendritic cells (pDCs). GS-9620 suppressed hepatitis B virus (HBV) in animal models of chronic infection and transiently activated HIV expression *ex vivo* in latently infected peripheral blood mononuclear cells (PBMCs) from virally suppressed patients. Currently, GS-9620 is under clinical evaluation for treating chronic HBV infection and for reducing latent reservoirs in virally suppressed HIV-infected patients. Here, we investigated the *in vitro* anti-HIV-1 activity of GS-9620. GS-9620 potently inhibited viral replication in PBMCs, particularly when it was added 24 to 48 h prior to HIV infection (50% effective concentration = 27 nM). Depletion of pDCs but not other immune cell subsets from PBMC cultures suppressed GS-9620 antiviral activity. Although GS-9620 was inactive against HIV in purified CD4^+^ T cells and macrophages, HIV replication was potently inhibited by conditioned medium derived from GS-9620-treated pDC cultures when added to CD4^+^ T cells prior to infection. This suggests that GS-9620-mediated stimulation of PBMCs induced the production of a soluble factor(s) inhibiting HIV replication in *trans*. GS-9620-treated PBMCs primarily showed increased production of interferon alpha (IFN-α), and cotreatment with IFN-α-blocking antibodies reversed the HIV-1-inhibitory effect of GS-9620. Additional studies demonstrated that GS-9620 inhibited a postentry event in HIV replication at a step coincident with or prior to reverse transcription. The simultaneous activation of HIV-1 expression and inhibition of HIV-1 replication are important considerations for the clinical evaluation of GS-9620 since these antiviral effects may help restrict potential local HIV spread upon *in vivo* latency reversal.

## INTRODUCTION

Innate immune responses typically involve a family of highly conserved pattern recognition receptors that include the membrane-based Toll-like receptors (TLRs), which detect distinct pathogen-associated molecular patterns (PAMPs) specific to infectious agents (1). There are at least 10 known members of this class of receptors in humans (named TLR1 to TLR10). Whereas TLR1, TLR2, TLR4, TLR5, and TLR6 are expressed at the cell surface, TLR3, TLR7, TLR8, and TLR9 are localized within intracellular vesicles, including the endoplasmic reticulum, endosomes, lysosomes, and endolysosomes. These intracellular TLRs recognize viral and microbial nucleic acids and, once they are activated, rapidly induce an immune response characterized by the production of acute-phase cytokines and antiviral factors (2).

Viruses containing single-stranded RNA genomes, including vesicular stomatitis virus (VSV), influenza A virus, and human immunodeficiency virus (HIV), are recognized by TLR7 and TLR8. TLR7 is highly expressed in plasmacytoid dendritic cells (pDCs) (3–5) but is also found to a lower extent on other leukocyte subpopulations, including monocytes (6), B cells (7), CD4^+^ T cells (8–10), and CD8^+^ T cells (8, 11, 12). Ligand binding to TLR7 results in activation of this receptor, recruitment of the cytoplasmic adaptor protein MyD88, and activation of the transcription factors nuclear factor kappa light chain enhancer of activated B cells (NF-κB) and interferon regulatory factor 7 (IRF7). These factors then translocate to the nucleus and trigger the production of various cytokines, including the type I interferons (IFNs) interferon alpha (IFN-α) and IFN-β, which act in both an autocrine and a paracrine fashion on infected and uninfected cells to produce an antiviral response. Binding of these proteins to the IFN receptors triggers the expression of numerous interferon-stimulated genes (ISGs) and the secretion of proinflammatory and immunomodulatory proteins with antiviral properties, such as protein kinase R, which inhibits viral protein synthesis, or the 2′,5′-oligoadenylate synthetase family, which degrades viral RNA, and in combination, their expression helps establish an antiviral state in the cell (13, 14).

Several molecules which bind TLR7 and induce immune responses leading to the control of HIV replication have been identified. These include the TLR7/8 agonist gardiquimod, which has been shown to induce the IFN-α that effectively inhibits HIV-1 replication *in vitro* in activated lymphocytes and macrophages at concentrations that activate TLR7 but not TLR8 (15). Activation of TLR7/8-mediated signaling pathways upon treatment with either single-stranded RNA or the small molecule TLR7/8 agonist resiquimod (R-848) greatly reduced the ability of lymphoid tissue to support HIV infection, with the stage of anti-HIV action likely to be after virus-host cell membrane fusion but before DNA integration into the host genome. TLR7/8 agonists also directly induced the release of HIV virions from latently infected monocytic cell lines, suggesting potential activation of the latent HIV reservoir (16). Thus, TLR7/8 triggering may have a dichotomous effect in HIV infection by preventing infection of CD4^+^ T cells while activating HIV expression in others.

GS-9620 is an orally bioavailable small-molecule TLR7-selective agonist (17, 18) with mean (50%) effective concentrations (EC_50_s) of 130 nM and 4,000 nM for TLR7- and TLR8-specific activation, respectively. It was recently reported that GS-9620 induced HIV RNA expression *ex vivo* in peripheral blood mononuclear cells (PBMCs) isolated from patients on suppressive antiretroviral therapy (ART) (19). In addition, multiple oral administrations of a close analog of GS-9620 in simian immunodeficiency virus-infected rhesus macaques in which virus was suppressed by ART induced transient plasma viremia, reduced the viral DNA content in lymphoid tissues, and established lower viral set points after ART cessation (20, 21). GS-9620 is currently in phase I clinical trials to evaluate the induction of the latent HIV reservoir and was found to be safe and well tolerated in patients with chronic hepatitis B virus (HBV) infection (22). Our current studies support the clinical evaluation of GS-9620, specifically focusing on the anti-HIV effects of the triggering of TLR7. We explore whether GS-9620-induced TLR7 activation could directly alter HIV-1 replication in PBMCs *in vitro* and the likely mechanisms involved in inhibition of HIV replication.

## RESULTS

### GS-9620 inhibits HIV-1 replication in activated PBMCs.

The antiviral activity of GS-9620 in isolated macrophages, CD4^+^ T cells, and total PBMCs was assessed following infection with replication-competent strain HIV-1_BaL_ or a single-cycle VSV G glycoprotein (VSV-G)-pseudotyped HIV-1 reporter virus encoding the luciferase protein (HIV-1_VSV-G-LUC_). In uninfected cultures, the viability of each cell type remained unchanged with GS-9620 up to 10 μM, the maximum concentration tested. As shown in Table 1, GS-9620 was inactive against HIV in isolated CD4^+^ T cells and macrophages up to the highest concentration tested (10 μM) but did show dose-dependent inhibition of HIV-1 replication in complete PBMCs following infection with either HIV-1_BaL_ (EC_50_ = 536 nM) or HIV-1_VSV-G-LUC_ (EC_50_ = 953 nM). Although GS-9620-mediated antiretroviral activity varied significantly between individual donors, its antiretroviral potency in PBMCs was enhanced by approximately 35-fold when GS-9620 was added 48 h prior to infection with HIV-1_VSV-G-LUC_ (EC_50_ = 27 nM). Similar potency improvements were also observed in GS-9620-pretreated PBMCs infected with HIV-1_BaL_ (data not shown). The antiretroviral activity was likely not due to cytotoxicity. A 50% cytotoxic concentration (CC_50_) value of 22 μM for uninfected activated PBMCs translates to a mean selectivity index (CC_50_/EC_50_ ratio) of 815-fold for GS-9620.

**TABLE 1:** *In vitro* anti-HIV-1 activity and cytotoxicity profiles of GS-9620 in primary cells

Target cell	EC_50_ (nM)^*a*^			CC_50_ (nM)^*b*^
	HIV-1_BaL_^*c*^	HIV-1_VSV-G-LUC_^*d*^	HIV-1_VSV-G-LUC_^*e*^	
Complete PBMCs	536 ± 830 (27)^*f*^	953 ± 1,115 (8)	27.2 ± 31.0 (21)	22,000 ± 5,200 (3)
Isolated CD4^+^ T cells	>10,000 (2)	>10,000 (3)	>2,000 (2)	>10,000 (5)
Isolated macrophages	>10,000 (2)	>7,300 (3)	>1,600 (2)	>10,000 (5)

a Data represent the mean ± SD EC_50_s obtained from triplicate measurements for 2 to 27 donors.

b Data represent the mean ± SD CC_50_ values obtained from triplicate measurements for 2 to 5 donors.

c GS-9620 treatment for 5 days postinfection (multicycle antiviral assay).

d GS-9620 treatment for 3 days postinfection (single-cycle antiviral assay).

e GS-9620 treatment for 2 days preinfection and 3 days postinfection (single-cycle antiviral assay).

f Data in parentheses represent the number of donors tested.

### GS-9620-derived anti-HIV activity is predominantly pDC dependent.

**FIG 1:**
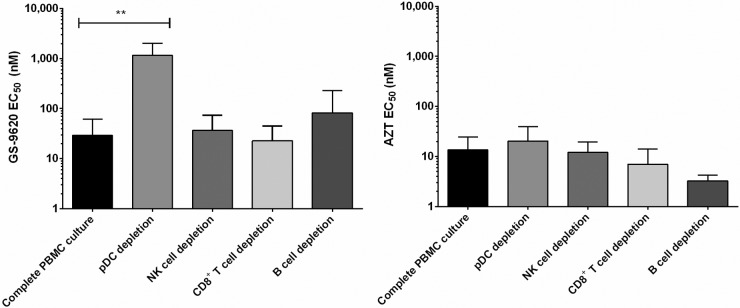
GS-9620-derived anti-HIV activity is predominantly pDC dependent. Mock-depleted or individual subset-depleted PBMC cultures were activated with PHA and IL-2 and then incubated with serially diluted GS-9620 or AZT (control) prior to infection with HIV-1_VSV-G-LUC_. Cultures were maintained in GS-9620-containing medium for 3 days, and EC_50_s were determined by quantifying luciferase expression. Mean EC_50_s ± SDs obtained from triplicate measurements in four independent donors are shown. *P* values were determined by the Mann-Whitney U test. **, *P* < 0.005.

Since GS-9620 is inactive directly against HIV-1 in macrophages and CD4^+^ T cells but highly potent against HIV-1 in complete PBMCs, we next investigated which specific cellular subsets were involved in eliciting GS-9620's antiviral effect in PBMCs. We compared total mock-depleted PBMCs and PBMC cultures individually depleted of CD8^+^ T cells, B cells, NK cells, or pDCs from each of four independent donors. Flow cytometry was used to confirm that the efficiency of cell type depletion was ≥95% in all cases. Cell cultures were then treated with GS-9620 for 48 h prior to infection with HIV-1_VSV-G-LUC_ and for the 3 days following infection with HIV-1_VSV-G-LUC_. While the antiviral activity observed was comparable between complete PBMCs and depleted cultures (mock, CD8^+^ T cell, B cell, and NK cell depletions), the depletion of pDCs significantly abrogated the GS-9620-induced antiviral effect (*P* = 0.003), increasing the EC_50_s 20- to 277-fold, depending on the donor being tested (Fig. 1, left). In contrast, the antiviral activity of zidovudine (AZT), a control HIV reverse transcriptase inhibitor, remained essentially unchanged across parallel complete and subset-depleted PBMC cultures (Fig. 1, right).

### A pDC-derived soluble factor(s) mediates GS-9620 antiviral activity in isolated HIV-infected CD4^+^ T cells.

**FIG 2:**
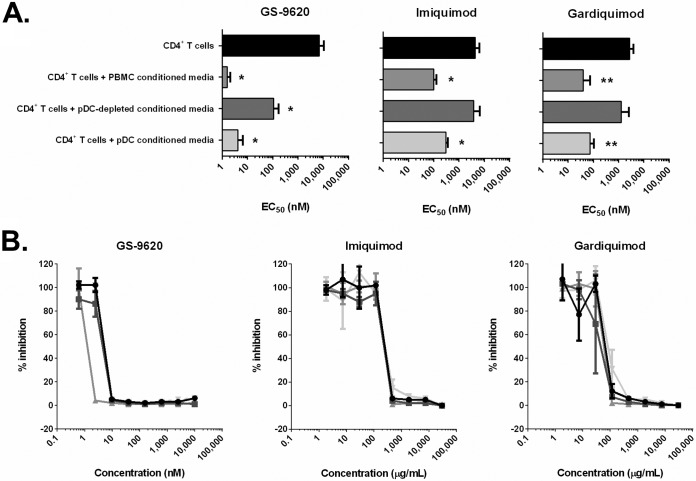
A pDC-derived soluble factor(s) mediates GS-9620 antiviral activity in isolated HIV-infected CD4^+^ T cells. (A) Antiviral activity was initially evaluated in isolated activated CD4^+^ T cell cultures that were treated with serially diluted GS-9620, imiquimod, or gardiquimod both before and after infection with HIV-1_VSV-G-LUC_ (black bars). To evaluate the potential role of soluble factors in TLR7 agonist-derived antiretroviral activity, GS-9620-, imiquimod-, and gardiquimod-conditioned supernatants from total PBMCs, pDC-depleted PBMCs, or isolated pDCs were transferred to activated CD4^+^ T cell cultures 24 h prior to infection with HIV-1_VSV-G-LUC_. EC_50_s were determined 3 days after infection by quantifying luciferase expression. Mean EC_50_s ± SDs obtained from triplicate measurements for four independent donors are shown. *P* values were determined by an unpaired *t* test. *, *P* < 0.05; **, *P* < 0.005. (B) Representative curves of antiviral activity (EC_50_) in HIV-infected CD4^+^ T cells following coculture with TLR7 agonist-conditioned supernatants from autologous pDCs obtained from four independent donors.

We next evaluated if a pDC-derived soluble factor(s) was involved in the anti-HIV effects of GS-9620. To do this, total PBMCs, pDC-depleted PBMCs, and pDCs isolated from four individual donors were independently treated with serially diluted GS-9620 for 36 h, and then cell-free culture supernatants were transferred to isolated autologous CD4^+^ T cells 24 h prior to infection with HIV-1_VSV-G-LUC_. Although direct GS-9620 treatment had no major effect on *de novo* HIV infection of isolated CD4^+^ T cells (EC_50_ > 7 μM), the addition of conditioned medium from GS-9620-treated PBMC cultures strongly inhibited (99%) *de novo* HIV infection in isolated CD4^+^ T cells (EC_50_ = 1.6 ± 0.5 nM), indicating that the antiviral activity of GS-9620 is mostly mediated by a soluble PBMC-derived antiviral factor(s) (Fig. 2). Likewise, the TLR7 agonists imiquimod and gardiquimod also showed no significant direct antiviral activity in autologous CD4^+^ T cells (EC_50_s = 9.4 ± 4.5 μM and 5.1 ± 0.4 μM, respectively; CC_50_s = 9.6 ± 1.7 μM and 7.0 ± 1.0 μM, respectively) but strongly suppressed HIV infection in isolated CD4^+^ T cells upon the addition of conditioned cell-free PBMC supernatants treated with imiquimod and gardiquimod (EC_50_s = 100 ± 30 nM and 40 ± 35 nM, respectively) (Fig. 2). Notably, the antiviral activity of each of these three TLR7 agonists observed in CD4^+^ T cells was significantly diminished following the transfer of cell-free supernatants from pDC-depleted PBMCs relative to that following the transfer of supernatants from complete PBMC cultures. Moreover, the addition of GS-9620-, imiquimod-, and gardiquimod-conditioned supernatants from isolated pDCs was found to be sufficient to achieve potent antiviral activity in CD4^+^ T cells (Fig. 2). These findings underscore the importance of pDC-derived soluble factors in GS-9620-, imiquimod-, and gardiquimod-dependent antiretroviral activity.

### GS-9620 predominantly induces interferon and IL-6 production in PBMC cultures.

**FIG 3:**
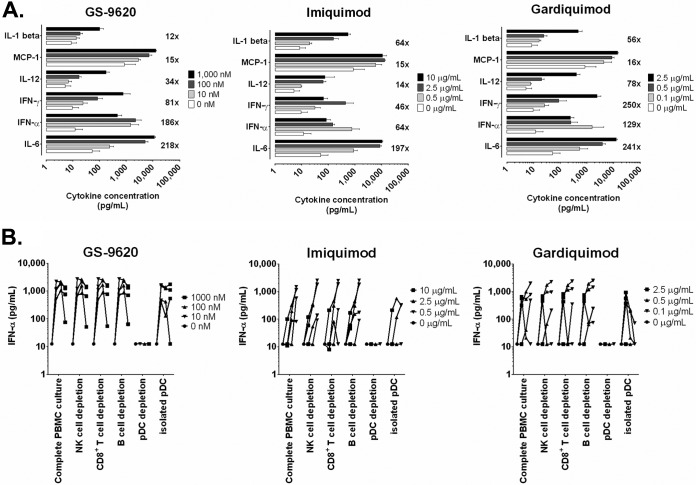
GS-9620 predominantly induces interferon and IL-6 production in complete PBMC cultures. (A) TLR7 agonist-induced cytokine levels observed in resting PBMCs at 36 h posttreatment with GS-9620 (10 to 1,000 nM), imiquimod (0.5 to 10 μg/ml), or gardiquimod (0.1 to 2.5 μg/ml) versus those observed in PBMCs treated with DMSO (vehicle control), as determined by Luminex analysis of cell-free culture supernatants. The top 6 of 25 total cytokines analyzed are shown for each TLR7 agonist. Data represent the mean values ± SDs obtained for four independent donors. Numbers represent the maximal mean fold increase in cytokine levels observed among the three tested compound concentrations. For each of the three TLR7 agonists, the levels of 11 analytes increased only 2- to 10-fold (IL-4, IL-8, IL-10, IL-1 receptor agonist, IL-2 receptor, IP-10, RANTES, monokine induced by IFN-γ, macrophage inflammatory proteins 1α and 1β, and eotaxin), whereas the levels of 8 analytes remained unchanged upon treatment with agonist (IL-2, IL-5, IL-7, IL-13, IL-15, IL-17, GM-CSF, and tumor necrosis factor alpha). (B) pDCs are necessary and sufficient for TLR7 agonist-induced IFN-α production. Cell-free supernatants were obtained from PBMC cultures (total and subset depleted) and isolated pDCs following a 36-h treatment with DMSO (vehicle control), GS-9620 (10 to 1,000 nM), imiquimod (0.5 to 10 μg/ml), or gardiquimod (0.1 to 2.5 μg/ml) and analyzed by IFN-α ELISA (lower limit of quantitation = 12.5 pg/ml). The IFN-α levels obtained from each of four independent donors at each of three test concentrations are shown.

To identify the potential soluble factor(s) involved in GS-9620-mediated antiviral activity, supernatants from PBMCs incubated with either dimethyl sulfoxide (DMSO; vehicle control) or TLR7 agonists (GS-9620, imiquimod, or gardiquimod) were analyzed at 36 h posttreatment for the production of cytokines and chemokines by a multiplex assay on a Luminex instrument (Fig. 3A). Relative to the levels in DMSO-treated cultures, GS-9620-conditioned supernatants showed the greatest fold increase in interleukin-6 (IL-6; 218-fold), IFN-α (186-fold), and IFN-γ (81-fold) levels. PBMCs treated with GS-9620 at 10 nM, a concentration closely approximating its observed anti-HIV EC_50_ in PBMCs, predominantly increased IFN-α production, with the mean IFN-α concentration being equal to 1,465 pg/ml. The levels of numerous additional chemokines and cytokines were also increased with GS-9620 treatment, including IL-12p40/p70 (34-fold), monocyte chemoattractant protein 1 (MCP-1; 15-fold), IL-1β (12-fold), and IL-10 (8-fold), but to a much lesser fold extent than those of IL-6 and type I interferons. A similar increase in the levels of the same six cytokines was seen with imiquimod- and gardiquimod-conditioned PBMC supernatants (Fig. 3A). Comparable levels of IFN-α were observed at each GS-9620 test concentration following treatment of complete and CD8^+^ T cell-, B cell-, and NK cell-depleted PBMC cultures; however, pDC-depleted PBMCs showed no notable increase in the levels of IFN-α production above the baseline measurements observed in DMSO-treated complete PBMC cultures (Fig. 3B). Moreover, high-level IFN-α production was observed in isolated pDCs treated with GS-9620, thus confirming that pDCs are the principal cellular source of IFN-α in response to GS-9620 treatment. Similar results were obtained with the TLR7 agonists imiquimod and gardiquimod, although both of these compounds showed somewhat higher interdonor variation than GS-9620 for the four donors tested herein. These findings suggest that IFN-α is the predominant soluble factor released by pDCs at GS-9620 concentrations associated with potent anti-HIV activity.

### IFN-α plays a dominant role in GS-9620 antiviral activity.

**FIG 4:**
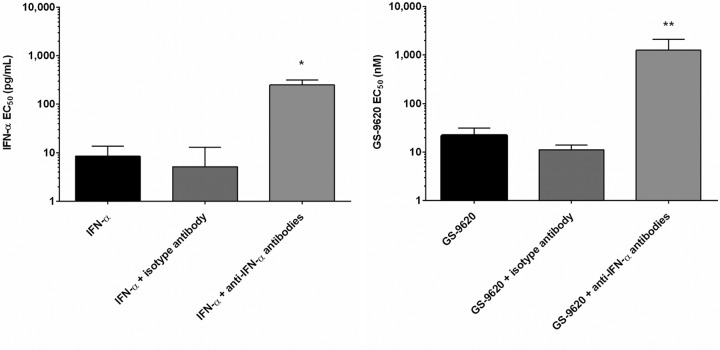
Blockade of IFN-α function reverses the HIV-1-inhibitory effect of GS-9620. Complete PBMCs were treated with recombinant IFN-α or GS-9620 with or without either IFN-α antibodies (neutralizing and antireceptor) or isotype control antibody for 24 h. Conditioned PBMC supernatant was then transferred to PHA-activated CD4^+^ T cells following a PHA washout. After a 24-h incubation, the CD4^+^ T cells were infected with HIV-1_VSV-G-LUC_ and redosed with IFN-α or GS-9620. Mean data ± SD obtained for at least three independent donors assayed in triplicate are shown. (Left) Recombinant IFN-α with or without IFN-α antibody treatments; (right) GS-9620 with or without IFN-α antibody treatments. *P* values were determined by an unpaired *t* test. *, *P* < 0.05; **, *P* < 0.005.

To determine whether the induction of IFN-α production by GS-9620 could protect against HIV-1 infection, we first measured the anti-HIV activity of recombinant IFN-α in the absence and presence of specific and isotype control antibodies. Consistent with its known antiviral properties, recombinant IFN-α was able to potently inhibit HIV replication in CD4^+^ T cells (EC_50_ = 8 ± 5 pg/ml, *n* = 5 donors). Moreover, the coadministration of antibodies specific to IFN-α and the IFN-α/β receptor (IFNAR) completely restored HIV replication in infected CD4^+^ T cell cultures up to the highest IFN-α concentration tested (*P* = 0.008), whereas cotreatment with an isotype control antibody had no notable effect on interferon antiviral activity (Fig. 4, left). These studies confirm that recombinant IFN-α protein is sufficient to inhibit HIV replication in CD4^+^ T cells. Notably, the addition of anti-IFN-α and anti-IFNAR antibodies, but not the isotype control antibody, also completely abolished the antiviral properties of supernatants derived from GS-9620-treated PBMCs on CD4^+^ T cells (*P* = 0.004) (Fig. 4, right). Taken together, these studies indicate a dominant role of GS-9620-induced IFN-α production in its anti-HIV activity.

### GS-9620 does not affect HIV entry into PBMCs.

**FIG 5:**
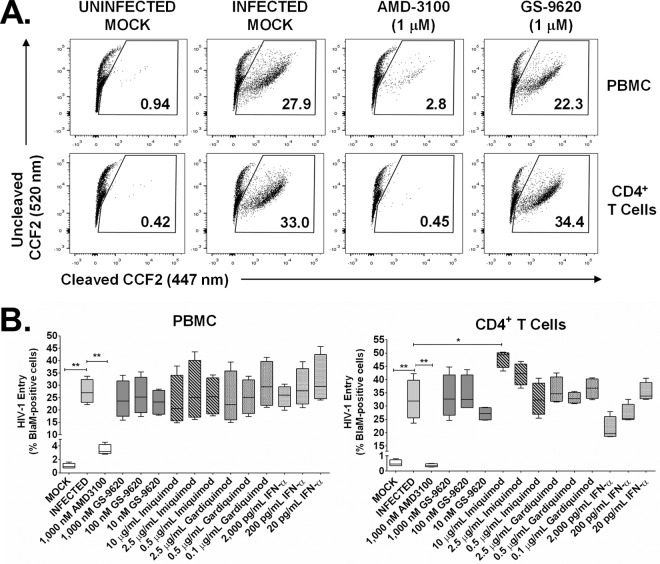
GS-9620 does not affect HIV entry into PBMCs or CD4^+^ T cells. After a 36-h pretreatment with DMSO (vehicle control), GS-9620, or control inhibitors (AMD3100, imiquimod, gardiquimod, or recombinant IFN-α protein), PBMCs and CD4^+^ T cells were infected with CXCR4-tropic HIV-1_NL4.3_ virions containing copackaged *vpr* protein covalently linked to the β-lactamase (BlaM-Vpr) enzyme or mock infected as a control. Transduced cells were then loaded with CCF2 reporter dye, a fluorescent substrate of β-lactamase, and the virus-cell membrane fusion was analyzed by flow cytometry to determine the percentage of cells containing intracellularly cleaved versus uncleaved CCF2 dye (447-nm and 520-nm emissions, respectively). Assays under each treatment condition were performed one or more times with each type of cell from four independent donors. (A) Representative flow cytometry plots from transduced PBMCs and CD4^+^ T cells obtained from a single donor are shown. (B) Complete HIV-1 entry data (minimum to maximum) obtained for transduced PBMCs and CD4^+^ T cells from four independent donors are shown. *P* values were determined by the Mann-Whitney U test. *, *P* < 0.05; **, *P* < 0.005.

The BlaM-Vpr assay was used to quantify HIV entry into cells treated with GS-9620. This assay uses HIV particles containing a copackaged β-lactamase enzyme which, when transferred to the target cell's cytosol following virus-host cell membrane fusion, can be detected and quantified through enzymatic cleavage of the fluorescent substrate CCF2 (23, 24). HIV-1_NL4.3_ containing BlaM-Vpr protein was used to infect PBMCs and CD4^+^ T cells from four donors following a 36-h pretreatment with GS-9620 or the control compounds. Target cells were loaded with CCF2 substrate dye, and CD3^+^ CD4^+^ CD8^−^ cells containing virus were enumerated by flow cytometry to detect the cleaved CCF2 dye after 16 h of incubation (Fig. 5A). Whereas only a low proportion of blue CD3^+^ CD4^+^ CD8^−^ cells was detected in PBMCs and CD4^+^ T cells following a mock infection (1.0% ± 0.4% and 0.5% ± 0.2%, respectively), mean rates of infection with HIV-BlaM were 27% ± 5% in PBMCs and 32% ± 7% in CD4^+^ T cells in the presence of DMSO (vehicle control) (Fig. 5B). As expected, treatment with the CXCR4 small-molecule control inhibitor AMD3100 (1 μM) greatly reduced the level of virus entry to near background levels in both cell types (Fig. 5B). In contrast, treatment with up to 1 μM GS-9620, equivalent to 37-fold its EC_50_ in PBMCs, did not significantly reduce the level of HIV entry in either cell type (Fig. 5B). Parallel treatments with imiquimod (≤2.5 μg/ml), gardiquimod (0.1 to 2.5 μg/ml), or recombinant IFN-α (20 to 2,000 pg/ml) also had no significant adverse effect on HIV entry into PBMCs or CD4^+^ T cells (Fig. 5B). These data indicate that the TLR7 agonists GS-9620, imiquimod, and gardiquimod must each induce a postentry block in HIV replication in PBMC cultures.

### GS-9620 inhibits HIV replication at or prior to the early stages of RT.

**FIG 6:**
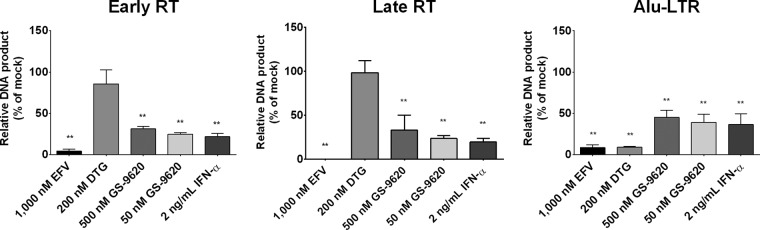
GS-9620 anti-HIV activity correlates with the inhibition of viral cDNA accumulation. Complete PBMCs were pretreated with DMSO (mock treatment), GS-9620 (50 to 500 nM), or control antiviral agents (EFV, DTG, or recombinant IFN-α protein) for 48 h prior to infection with HIV-1_BaL_. Cells were maintained in compound-containing medium postinfection, and total DNA was harvested 24 h (early and late RT) and 72 h (Alu-LTR) later. The relative amounts of early (minus-strand, strong-stop, and 1st-strand-transfer DNA) and late (2nd-strand-transfer DNA) reverse transcription and integration (Alu-LTR) products were quantified by qPCR and ddPCR, respectively. Shown are mean ± SD values for early RT, late RT, and Alu-LTR products normalized to the values for DMSO-treated controls obtained from 3 independent experiments performed in triplicate. *P* values were determined by the Mann-Whitney U test. **, *P* < 0.005.

To define the postentry molecular defects in HIV replication associated with GS-9620 treatment, PBMCs were first treated with DMSO (vehicle control), GS-9620 (50 nM or 500 nM), or control antiviral agents (1 μM efavirenz [EFV], 200 nM dolutegravir [DTG], or 2 ng/ml recombinant IFN-α protein) for 48 h prior to infection with HIV-1_BaL_. Cells were maintained in compound-containing medium postinfection, and total DNA was harvested 24 h and 72 h later. The amounts of reverse-transcribed strong-stop and 2nd-strand-transfer DNA products (early and late reverse transcription [RT], respectively) and integration products (composed of a genomic Alu repeat element and a neighboring integrated HIV long terminal repeat [LTR]) were quantified by quantitative PCR (qPCR) and droplet digital PCR (ddPCR), respectively, and then the levels were normalized to those for the DMSO-treated controls (Fig. 6). As expected, treatment with the reverse transcription control inhibitor EFV significantly reduced the levels of accumulation of all three replication products, whereas treatment with the integrase strand transfer control inhibitor DTG specifically reduced only integration products without noticeably changing early/late reverse transcription product formation. Notably, PBMCs treated with either GS-9620 or IFN-α produced a product accumulation pattern similar to that observed with PBMCs treated with EFV, indicating that both GS-9620 and IFN-α inhibit HIV replication in PBMCs at or prior to the early stages of reverse transcription.

## DISCUSSION

Members of the TLR family of pattern recognition receptors play an important role in innate immune responses to viral and bacterial infections. Activation of TLR7 signaling pathways ultimately leads to the release of antiviral and proinflammatory cytokines, such as type I interferons (IFN-α and IFN-β), that help the host rapidly mount an immune response to infection with an invasive pathogen. Agonists to TLR7 are attractive therapeutic agents with the potential of targeting innate and acquired immunity in fighting viral infections (22, 25). In this study, we investigated the ability of GS-9620, a potent and selective oral small-molecule agonist of TLR7, to inhibit HIV replication *in vitro* in total PBMCs and cell cultures depleted of specific immune cell subsets.

GS-9620 showed potent antiviral activity in human PBMCs acutely infected with HIV-1, and this activity was especially enhanced when it was added 24 to 48 h prior to the infection. Although GS-9620 was inactive against HIV in purified CD4^+^ T cells and macrophages, the coincubation of isolated CD4^+^ T cells with conditioned medium derived from PBMCs pretreated with GS-9620 24 h prior to infection resulted in a potent antiviral response. Further investigation using a cell depletion and subset isolation approach revealed that pDCs were necessary and sufficient for GS-9620-mediated anti-HIV activity. These findings, which were further replicated with two additional TLR7 agonists (imiquimod and gardiquimod), are consistent with previous reports implicating pDCs as the initial effector cells in the antiviral response linked to TLR7 activation, namely, via increased production of IFN-α (26, 27), and are in contrast to a recent report showing that both imiquimod and gardiquimod promote HIV infection of CD4^+^ T cells (28). Conversely, whereas B cells have been implicated in IFN-α-mediated anti-HIV activity and a role for natural killer (NK) cells and CD8^+^ T cells has been linked to TLR7/8-mediated anti-HIV activity (11), our results indicate that these cells are individually dispensable for the IFN-α-mediated anti-HIV effects observed upon GS-9620 treatment. Moreover, our finding that GS-9620-conditioned medium from complete PBMCs but not pDC-depleted cultures could potently inhibit HIV replication in isolated CD4^+^ T cells demonstrates that GS-9620-induced antiviral activity against HIV does not require cell-cell contact. It also indicates that a GS-9620-induced pDC-derived soluble factor(s) is sufficient for HIV-1 inhibition.

The levels of a wide array of cytokines and chemokines were selectively increased in GS-9620-conditioned PBMC supernatants relative to those in vehicle-treated cultures. Among these was IFN-α, whose level in PBMCs from healthy donors increased 186-fold upon GS-9620 treatment, irrespective of mitogenic stimulation. Induced IFN-α production was not detected in pDC-depleted PBMC cultures treated with GS-9620, and these conditioned supernatants also failed to inhibit HIV replication in cocultured CD4^+^ T cells. Conversely, IFN-α production was similarly enhanced in isolated pDC cultures upon GS-9620 treatment, and these conditioned supernatants were sufficient to inhibit HIV replication in cocultured CD4^+^ T cells. In addition, when neutralizing antibodies against soluble IFN-α and IFN receptors were included, GS-9620-conditioned medium from total PBMC cultures lost its antiviral activity in infected CD4^+^ T cells. Thus, our data indicate that upon GS-9620 treatment, pDCs play an essential role in constraining HIV infection via the increased production and release of soluble IFN-α.

The mechanism of IFN-α-mediated inhibition of HIV replication has been previously studied in a variety of experimental settings and cell types and is most commonly associated with an early-stage, postentry block in the viral life cycle, resulting in reduced viral cDNA accumulation (24, 29–32). The observation of potent antiviral activity against single-cycle VSV-G-pseudotyped HIV-1 particles following treatment with recombinant IFN-α or GS-9620-conditioned PBMC supernatants is consistent with this mechanistic model and demonstrates independence from the activities of both the cellular entry receptors and the HIV-1 Env protein. Consistent with this notion, our quantitative flow cytometry-based measurements showed that GS-9620 treatment does not inhibit HIV entry into primary cells. Quantitative assessment of the postentry stages of HIV-1 replication further showed a strong inhibitory effect on the accumulation of nascent viral cDNAs (and downstream integration products) in GS-9620-treated PBMCs. Interestingly, this replication defect was similar to that observed in PBMCs treated with recombinant IFN-α protein. Although the replication defect was less profound than that induced by the reverse transcriptase inhibitor efavirenz, antiviral activity data obtained from studies using HIV-1_VSV-G-LUC_ underscore that this is a predominant mechanism of GS-9620-mediated inhibition of HIV-1. This suggests that GS-9620-mediated TLR7 activation results in the production of a pDC-derived soluble factor(s), especially IFN-α, that contributes to the blockade of HIV infection postentry at or prior to the reverse transcription step. It remains to be determined whether the GS-9620-induced replication block is mediated through direct effects on components of the reverse transcription complex (15) or through the increased turnover of viral RNA prior to the reverse transcription step (24).

The antiviral properties associated with TLR7 activation in mammalian cells have been described by several groups using distinct small-molecule TLR7/8 agonists. Notably, the TLR7/8 agonist gardiquimod inhibited HIV replication in macrophages and activated PBMCs at concentrations that specifically activate TLR7 signaling. This molecule also induced IFN-α production, and its induced anti-HIV effects were reversed by blockage of the IFN-α receptor (15). The dual specificity of the TLR7/8 agonist resiquimod (R-848) induced the production of a soluble factor(s) with anti-HIV activity in primary cells. Both gardiquimod and resiquimod induced a postentry preintegration block in HIV replication that was significantly enhanced upon exposure to these TLR agonists prior to HIV infection. Our findings obtained using the TLR7-selective agonist GS-9620 are in close agreement with the observations described above. However, in contrast to our findings, the resiquimod study by Schlaepfer et al. implicated B cells, rather than pDCs, as the primary TLR7/8-responsive cell type and concluded that the sum total of cytokine changes induced by resiquimod, rather than IFN-α in particular, was primarily responsible for the resiquimod-induced antiviral effect (16). Although the exact nature of these mechanistic differences is not clear, the authors suggest that the coactivation of TLR8-specific signaling pathways associated with resiquimod treatment may be an important contributing factor. Further studies by this group substantiated this theory and demonstrated TLR8-mediated HIV activation from latently infected cells via induced TNF-α acting in an autocrine and paracrine fashion (33), suggesting a promising strategy for attacking the latent HIV reservoir in patients on antiretroviral therapy. In agreement with this theory, GS-9620 is under investigation for HIV latency reactivation *in vitro* (19) and *in vivo* (20, 21).

Collectively, these data indicate that innate immune modifiers, such as TLR agonists, likely strike a fine balance between induced antiviral effects and the establishment of a pro-HIV state due to immune activation and activation of HIV expression. We propose a model where, in an ART-suppressed HIV-infected individual, localized innate immune responses triggered by GS-9620, either alone or in combination with other agents, could help reactivate expression of the latent HIV reservoir, followed by the killing of infected cells through viral cytopathic effects and/or host immune responses. The antiviral activity observed with GS-9620 could help further restrict the potential local spread of the virus to new target cells as part of a kick-and-kill intervention strategy aimed at reducing HIV reservoirs in virally suppressed HIV-infected patients.

## MATERIALS AND METHODS

### Reagents.

GS-9620 was synthesized at Gilead Sciences, Inc. (Foster City, CA, USA), as previously described (18). The small-molecule inhibitors of HIV reverse transcription (zidovudine [AZT], efavirenz [EFV]) and HIV integration (dolutegravir [DTG]) were purchased from Toronto Research Chemicals (Toronto, Ontario, Canada) and Shanghai Medicilon Inc. (Shanghai, China), respectively. AMD3100, a small-molecule inhibitor of HIV-1 entry via the CXCR4 coreceptor, as well as the cytotoxicity control compound puromycin, were each purchased from Sigma (St. Louis, MO, USA). All five compounds were individually reconstituted in DMSO and stored frozen at −20°C. The small-molecule TLR7 agonists imiquimod and gardiquimod were purchased from InvivoGen (San Diego, CA, USA), reconstituted in water, and stored frozen at −20°C. Recombinant interferon alpha (IFN-α) protein and monoclonal antibodies against human IFN-α (MMHA-2) or human IFN-α/β receptor chain 2 (MMHAR-2) were purchased from PBL Assay Science (Piscataway, NJ, USA) and stored in single-use aliquots at −80°C. Purified mouse IgG2a(κ) was used as an isotype control antibody (BioLegend, San Diego, CA). Peridinin chlorophyll protein-Cy5.5 mouse anti-human CD3, BV711 mouse anti-human CD4, and allophycocyanin-H7 mouse anti-human CD8 antibodies, used for flow cytometry experiments, were purchased from BD Biosciences (San Jose, CA, USA) and stored at 4°C in the dark.

### Primary cells.

Leucopacks were purchased from AllCells (Alameda, CA, USA) and were obtained from consenting healthy volunteers participating in a LeukoLab institutional review board (IRB)-approved donor program. Donors were negative for HIV-1, hepatitis B virus, and hepatitis C virus infections. Human PBMCs were isolated from fresh leucopacks by a standard Ficoll-Hypaque (Amersham, Piscataway, NJ, USA) gradient centrifugation technique. Primary human CD4^+^ T cells, monocytes, and pDCs were isolated from PBMCs by negative selection using the magnetically labeled antibody cocktails provided, respectively, in EasySep Human CD4^+^ T cell, human monocyte, and human plasmacytoid DC enrichment kits (Stemcell Technologies, Vancouver, BC, Canada). Individual PBMC subsets (pDCs, CD8^+^ T cells, NK cells, and B cells) were depleted by positive selection using magnetically labeled antibody cocktails provided in EasySep human custom pDC, human CD8, human CD56, and human CD19 positive-selection kits, respectively (Stemcell Technologies). Mock-depleted PBMC cultures were prepared in parallel using the same procedure described above, except that the addition of antibody was excluded. Cells were cultured in RPMI 1640 cell culture medium (Life Technologies, Grand Island, NY) supplemented with 10% fetal bovine serum (FBS), 2 mM glutamine, and 50 units/ml penicillin plus 50 μg/ml streptomycin (complete RPMI). PBMC cultures (complete and subset-depleted cultures) and CD4^+^ T cells were activated for 48 h at 37°C by addition of 1 μg/ml phytohemagglutinin (PHA; Sigma) and 5 ng/ml (50 units/ml) interleukin-2 (IL-2; Roche Diagnostics, Indianapolis, IN). Monocytes were incubated in complete RPMI cell culture medium supplemented with 10% FBS, 10% human serum (Sigma), and 50 ng/ml granulocyte-macrophage colony-stimulating factor (GM-CSF; R&D Systems, Minneapolis, MN, USA) to enable differentiation into monocyte-derived macrophages (MDMs) over a period of 8 days. The purity of the CD4^+^ T lymphocytes, monocytes, and differentiated macrophages was confirmed by fluorescence-activated cell sorter staining using anti-CD4, anti-CD14, and anti-CD11c antibodies (Becton Dickinson, Franklin Lakes, NJ, USA), respectively.

### Plasmids and virus stocks.

HIV-1_BaL_ (Advanced Biotechnologies, Columbia, MD, USA) was serially passaged in human PBMCs and used to infect CD4^+^ T cells and macrophages for antiviral activity assessments. Plasmid pKS13ΔEnv is an NL4.3-based construct that expresses a pGL3 firefly luciferase reporter gene (Promega, Madison, WI, USA) in place of the coding frame of the *nef* gene and contains frameshift mutations to knock out *vpr* and *env* expression. The pHCMV-G expression plasmid expressing the vesicular stomatitis virus G glycoprotein (VSV-G) envelope protein has been previously described (34). The pCMV-BlaM-Vpr plasmid expressing the β-lactamase–*vpr* fusion protein has been previously described (35). To prepare VSV-G-pseudotyped reporter virus stocks (HIV-1_VSV-G-LUC_), subconfluent HEK293T cells were transfected with a 3:1 molar ratio of plasmids pKS13ΔEnv and pHCMV-G using Lipofectamine 2000 (Invitrogen, Carlsbad, CA, USA). HIV virions containing the BlaM-Vpr chimera (HIV-BlaM) were produced by cotransfecting subconfluent HEK293T cells with the infectious molecular HIV-1 clone pNL4.3 and pCMV-BlaM-Vpr. Viral supernatants were collected at 48 h posttransfection, concentrated 20-fold using VivaSpin 100,000-molecular-weight-cutoff columns (Sartorius, Göttingen, Germany), and stored at −80°C in single-use aliquots. The viral inocula were normalized on the basis of the p24 content using a commercially available enzyme-linked immunosorbent assay (ELISA) kit (PerkinElmer, Waltham, MA, USA).

### Antiviral assays.

Antiviral assays were performed in two variations, namely, by treatment of cell cultures with compounds immediately following infection with HIV-1 or by the additional inclusion of a 48-h compound pretreatment prior to infection. Human PBMCs and CD4^+^ T cells from each donor were independently infected in bulk using HIV-1_BaL_ (10 ng p24 equivalent virus input per 10^6^ cells) by gently rocking the cultures in the presence of virus for 3 h at 37°C. Cells were then pelleted by centrifugation at 500 × *g* for 5 min, washed twice with complete RPMI to remove any unadsorbed virus, and then seeded into 96-well tissue culture plates by adding 2 × 10^5^ cells per well in 100 μl. Eight-point 4-fold serial dilutions of GS-9620 or control compounds were made in complete RPMI containing 50 units/ml IL-2 and added in triplicate to wells containing cells (100 μl per well). The cultures were incubated in a 5% CO_2_ incubator at 37°C for 6 days, after which cell-free culture supernatants were harvested and the amount of HIV was quantified by the p24 antigen ELISA (PerkinElmer) according to the manufacturer's protocol. Alternatively, freshly isolated PBMCs or CD4^+^ T cells from each donor were seeded in solid white 96-well flat-bottom Costar plates (1 × 10^6^ per well; Corning, NY, USA) in complete RPMI containing PHA and IL-2 and incubated at 37°C for 48 h. Cells were infected using HIV-1_VSV-G-LUC_ (10 ng p24 equivalent virus input per 10^6^ cells) by spinoculation at 1,200 × *g* for 2 h at room temperature. Following infection, the virus-containing medium was removed and medium containing 50 units/ml IL-2 and serially diluted compound was added in triplicate. At 3 days postinfection, 100 μl One-Glo luciferase substrate (Promega) was added to each well and the luminescence signal (in relative light units [RLUs]) was measured using an Envision plate reader (PerkinElmer). To assess the effect of GS-9620 treatment prior to infection with HIV, individual target cells (i.e., CD4^+^ T cells, total PBMCs, pDCs, or subset-depleted PBMCs) were incubated with serially diluted compound or recombinant IFN-α protein in triplicate for 48 h in the presence of PHA and IL-2 prior to spinoculation with HIV-1_VSV-G-LUC_. Infected cells were maintained in compound- or IFN-α-containing medium for 3 days, followed by RLU measurement using the One-Glo substrate. Adherent MDMs were prepared in 96-well Costar plates (3 × 10^5^ per well) by differentiating monocytes using 50 ng/ml GM-CSF and 10% human serum. MDMs were then infected with HIV-1_BaL_ (5 ng p24 equivalent virus input per 10^6^ cells) for 4 h in 50 μl RPMI, washed twice with RPMI, and incubated with 200 μl complete RPMI containing 50 ng/ml GM-CSF and serially diluted compounds. MDM cultures received one change of drug-containing medium on day 6 and were incubated for a total of 13 days at 37°C, after which time the supernatants were harvested and the amount of HIV produced was quantified by the p24 ELISA (PerkinElmer). Alternatively, MDMs were pretreated or not with serially diluted compounds in triplicate for 48 h, infected with HIV-1_VSV-G-LUC_ (3 ng p24 equivalent virus input per 10^6^ cells) by incubation at 37°C for 4 h, and redosed with compound for 3 days, and luciferase expression was quantified as described above.

To assess the antiviral effect of TLR7 agonist-conditioned medium, unstimulated and noninfected PBMCs and pDC-depleted PBMC cultures were individually incubated in 96-well flat-bottom Costar plates (1 × 10^6^ cells per well) in complete RPMI in triplicate with serially diluted GS-9620, imiquimod, or gardiquimod at 37°C for 36 h. Isolated pDCs were also treated similarly but were seeded at 1 × 10^4^ cells per well. Autologous CD4^+^ T cells were isolated in parallel, activated in bulk using PHA and IL-2 at 37°C for 24 h, and then seeded into solid white 96-well flat-bottom Costar plates (3 × 10^5^ per well) in complete RPMI following PHA washout. At this point, supernatants from TLR7 agonist-conditioned PBMC, pDC, and pDC-depleted PBMC cultures were harvested and transferred into the 96-well plates containing the autologous CD4^+^ T cells. After a 24-h coincubation, the conditioned medium was removed and the CD4^+^ T cells were infected by spinoculation using HIV-1_VSV-G-LUC_ and assayed for antiviral activity as described above.

Data analysis for the antiviral activity in all cell types was performed using XLfit software (IDBS, Guildford, Surrey, UK) to calculate EC_50_s from an 8-point dose-response curve.

### Cytotoxicity assays.

GS-9620, imiquimod, gardiquimod, and puromycin (control) cytotoxicity was assessed in primary human PBMC, CD4^+^ T cell, and macrophage cultures. Uninfected PBMCs and CD4^+^ T cells from individual donors were first activated in bulk using PHA and IL-2 at 37°C for 48 h and then seeded into 96-well plates at a density of 2 × 10^5^ cells per well, before being dosed in triplicate with 4-fold serial dilutions of compounds prepared in RPMI cell culture medium. Monocyte-derived macrophage cultures were prepared as described above and dosed similarly with serial dilutions of compound. Cells were incubated in a 5% CO_2_ incubator at 37°C for a period of 5 days (PBMCs and CD4^+^ T cells) or 13 days (macrophages). Macrophage cultures received a single change of drug-containing medium on day 6 of the assay. At the end of the assay, cell viability was assessed using the luminescent readout of the CellTiter-Glo assay (Promega), performed according to the manufacturer's recommendations, after normalization of the readout to that for the no-drug (DMSO-treated) control wells. Data analysis for the cytotoxicity measurements for each cell type was performed using XLfit software (IDBS). Mean CC_50_ values were calculated from triplicate measurements using an 8-point dose-response curve obtained in at least three independent experiments.

### Cytokine measurements.

Cell-free culture supernatants were obtained from 8 × 10^5^ complete PBMCs, 8 × 10^3^ pDCs, and 8 × 10^5^ PBMCs depleted of individual PBMC subsets (pDCs, CD8^+^ T cells, NK cells, and B cells) after incubation for 36 h in the absence (control) or presence of GS-9620 (1,000, 100, or 10 nM), imiquimod (10, 2.5, or 0.5 μg/ml), or gardiquimod (2.5, 0.5, or 0.1 μg/ml). All samples were quantified using a human IFN-α ELISA kit (R&D Systems), which has a lower limit of quantitation equal to 12.5 pg/ml. Samples from complete PBMCs, pDC-depleted PBMCs, and isolated pDCs were further analyzed for changes in the levels of 25 cytokines compared to those achieved with DMSO (vehicle control) treatment using a multiplex assay (Life Technologies) on a Luminex instrument.

### Virus entry assay.

The virus-host cell membrane fusion assay was performed essentially as described previously (23, 24). Briefly, human PBMCs or CD4^+^ T cells from each of four independent donors were seeded in 96-well Costar plates (1 × 10^6^ per well) and treated with GS-9620, imiquimod, gardiquimod, recombinant IFN-α protein, AMD3100, or DMSO (vehicle control) for 48 h. Cell cultures were then infected by spinoculation at 1,200 × *g* for 2 h with HIV-BlaM (400 ng p24 equivalent virus input per 10^6^ cells) containing copackaged *vpr* protein covalently linked to the β-lactamase enzyme, followed by a 1-h incubation at 37°C. Cells were washed in CO_2_-independent medium (Gibco, Rockville, MD, USA) and then loaded with CCF2-AM dye for 1 h at room temperature per the manufacturer's protocol (Life Technologies). Cells were washed twice with CO_2_-independent medium and resuspended in 200 μl of CO_2_-independent medium containing 10% FBS and 2.5 mM probenecid (Sigma) before the β-lactamase reaction was allowed to develop for 16 h at room temperature in the dark. Cells were washed once in phosphate-buffered saline (PBS) before immunostaining for 20 min at room temperature in the dark. Cells were washed once with PBS, fixed in a 4% solution of paraformaldehyde for 20 min at room temperature in the dark, and washed again. The change in the emission fluorescence of the CCF2-AM dye after cleavage by cytoplasmic BlaM-Vpr protein was measured in CD3^+^ CD4^+^ CD8^−^ cells using an LSRFortessa flow cytometer (Becton Dickinson). Green (520 nm, uncleaved CCF2) and blue (447 nm, cleaved CCF2) emissions were detected using HQ545/90 and HQ455/50 filters (BD Biosciences), respectively. Data were analyzed using FlowJo software (TreeStar, Ashland, OR, USA).

### Quantification of HIV DNA.

PBMCs from two independent donors were seeded in triplicate in 6-well Costar plates (4 × 10^6^ per well) and treated with GS-9620, EFV, DTG, recombinant IFN-α, or DMSO (vehicle control) for 48 h at 37°C. Cells were infected with HIV-1_BaL_ (100 ng p24 equivalent virus input per 10^6^ cells) by spinoculation at 1,200 × *g* for 2 h, followed by a 1-h incubation at 37°C. Cells were washed, resuspended in compound-containing medium, and harvested at 24 h for early and late RT product quantification and at 72 h for Alu-LTR integration product quantification. Cell pellets were stored at −80°C until viral DNA isolation using a QIAamp DNA minikit (Qiagen, Valencia, CA, USA) and quantified using TaqMan real-time PCR and an ABI Prism 7900HT sequence detection system (Applied Biosystems, Foster City, CA, USA) or droplet digital PCR (ddPCR) using a QX200 ddPCR system (Bio-Rad, Hercules, CA, USA). The conventional primers and probes used for qPCR detection of specific minus-strand viral DNA products corresponding to early (RU5 and U3) and late (GAG and PBS) stages of reverse transcription and endogenous cellular β-globin have been described previously (36–39). All primers and labeled probes were synthesized by Integrated DNA Technologies, Inc. (San Diego, CA, USA). For each experiment, a standard curve of the concentration of each amplicon from 10^3^ to 10^8^ linearized copies of each vector plus a no-template control, all diluted into an equivalent amount of 500 ng of sheared salmon sperm DNA (Life Technologies), was prepared. The amount of each amplicon from each extracted DNA treatment sample was measured in triplicate. Quantitative real-time PCR mixtures contained 1× Roche LightCycler 480 probe master mix (Roche Diagnostics, Indianapolis, IN, USA), 300 nM forward primers, 300 nM reverse primers, 100 nM probe primers, and 500 ng of DNA in a 25-μl volume. After initial incubations at 50°C for 2 min and 95°C for 10 min, 40 cycles of amplification at 15 s at 95°C were carried out, followed by 1 min at 60°C. Copy numbers were normalized by endogenous β-globin levels and represented as a percentage of the total copy number of the no-drug-infected control (set to 100%) for each amplicon measured.

### Statistical analysis.

GraphPad Prism software (La Jolla, CA, USA) was used for statistical analysis. An unpaired two-tailed *t* test was used for comparisons between two groups. For nonparametric analysis of two groups, a Mann-Whitney U test was performed. In both cases, a *P* value of 0.05 or less was considered significant.
